# The intratumour microbiota and neutrophilic inflammation in squamous cell vulvar carcinoma microenvironment

**DOI:** 10.1186/s12967-023-04113-7

**Published:** 2023-04-28

**Authors:** Natalia Rustetska, Magdalena Szczepaniak, Krzysztof Goryca, Elwira Bakuła-Zalewska, Małgorzata Figat, Artur Kowalik, Stanisław Góźdź, Magdalena Kowalewska

**Affiliations:** 1 Department of Molecular and Translational Oncology, Maria Sklodowska-Curie National Research Institute of Oncology, 02-781 Warsaw, Poland; 2 Department of Molecular Diagnostics, Holycross Cancer Centre, 25-734 Kielce, Poland; 3 Genomics Core Facility, Centre of New Technologies, University of Warsaw, 02-097 Warsaw, Poland; 4 Department of Pathology, Maria Sklodowska-Curie National Research Institute of Oncology, 02-781 Warsaw, Poland; 5 Department of Gynecologic Oncology, Maria Sklodowska-Curie National Research Institute of Oncology, 02-097 Warsaw, Poland; 6 Division of Medical Biology, Institute of Biology, Jan Kochanowski University, 25-406 Kielce, Poland; 7 Department of Clinical Oncology, Holycross Cancer Centre, 25-734 Kielce, Poland; 8 Collegium Medicum, Jan Kochanowski University, 25-317 Kielce, Poland

**Keywords:** Vulvar carcinoma, Microbiota, *Fusobacterium nucleatum*, *Pseudomonas aeruginosa*, Neutrophil serine protease, Microabscess

## Abstract

**Background:**

A causal link between microbiota composition (dysbiosis) and oncogenesis has been demonstrated for several types of cancer. Neutrophils play a role in both immune protection against bacterial threats and carcinogenesis. This study aimed to characterise intratumoral bacteria in vulvar squamous cell carcinoma (VSCC) and their putative effect on neutrophil recruitment and cancer progression.

**Methods:**

Clinical material was obtained from 89 patients with VSCC. Next-generation sequencing (NGS) of 16S rRNA and quantitative polymerase chain reaction (qPCR) were used to detect bacterial species in VSCC. To verify neutrophil activation, CD66b expression in tumour specimens was analysed by immunohistochemistry (IHC). Subsequently, IHC was applied to detect the main neutrophil serine proteases (NSPs), cathepsin G (CTSG), neutrophil elastase (ELANE), and proteinase 3 (PRTN3) in VSCC.

**Results:**

*Fusobacterium nucleatum* and *Pseudomonas aeruginosa* were identified as tumour-promoting bacteria, and their presence was found to be associated with a shorter time to progression in VSCC patients. Furthermore, high abundance of CD66b, the neutrophil activation marker, in VSCC samples, was found to relate to poor survival of patients with VSCC. The selected NSPs were shown to be expressed in vulvar tumours, also within microabscess. The increased numbers of microabscesess were correlated with poor survival in VSCC patients.

**Conclusions:**

Our results show that neutrophilic inflammation seem to be permissive for tumour-promoting bacteria growth in VSCC. The findings provide new therapeutic opportunities, such as based on shifting the balance of neutrophil populations to those with antitumorigenic activity and on targeting NSPs produced by activated neutrophils at the inflammation sites.

**Supplementary Information:**

The online version contains supplementary material available at 10.1186/s12967-023-04113-7.

## Background

The human body is inhabited by the microbiota, that is, bacterial, viral, and fungal populations composed of approximately 30 trillion microbes, with the majority residing in the gut. Gut colonization begins with vertical transmission through ingestion of maternal microorganisms by new-borns during vaginal births. More microorganisms are transferred by breastfeeding, moreover, maternal milk provides substances promoting mutualist microbes and restricting the growth of harmful microbes in the infant [1]. The origin of the microorganisms that populate the genital tract has been traced to the rectum [2]. It is hypothesized that the vulva may constitute a transition zone, from cutaneous commensals to the vaginal microbiome with intestinal influences [3]. The interactions of the microbiota with host cells within the body ecosystems are critical for human health by affecting multiple physiological functions, including interactions of microbes with the immune system that shape host immunity [4].

Dysbiosis, which occurs when an imbalance in the composition of the microbiota results in overproliferation of harmful microbes, is associated with the pathogenesis of several diseases, including cancer [1, 5]. The microbiota can confer a carcinogenic effect by promoting local oncogenic inflammation or systemic metabolic and immune dysregulation [5]. The International Agency for Cancer Research designated 11 microorganisms—including human papillomavirus (HPV)—as carcinogens [6], and these are known to contribute to approximately 15% of cancers [7]. However, it seems the significance of microorganisms may be underestimated, as in dysbiosis, due to disrupted cooperation between normal cells and beneficial microbes, the entire microbial ecosystems may enhance cancer initiation and progression [8]. Different compositions of the intratumour microbiota were revealed in various types of tumours, including breast tumours that harbour a rich and diverse microbiota [9], although breast cancer is commonly considered to arise from aseptic tissue.

The healthy skin [10] and female reproductive tract are inhabited by microbe communities [11], and these microbiota were examined in detail. However, the microbiota of the vulva, being a transition between cutaneous epithelium of the skin and the mucosa of the female urogenital tract, remained essentially unexplored until a report by Vongsa et al. [12]. According to this study, the vulvar microbiota is dominated by the *Lactobacillus* flora. However, there are no specific data on the microbial tenants in vulvar carcinoma examining the context of vulvar carcinogenesis. Previously, we have identified inflammation as a major driver of the progression of vulvar carcinoma and hypothesized that microflora disturbances may be the causative factor in vulvar cancer-related inflammation [13]. In this study, our objective was to characterize intratumoral bacteria in vulvar carcinoma using next generation sequencing of 16S rRNA.

## Methods

### Patients

Clinical material was obtained from 89 randomly selected patients treated for primary vulvar squamous cell carcinoma (VSCC) (median age 70.8 years, rage 37.1–94.2) at the Maria Sklodowska-Curie National Research Institute of Oncology in Warsaw, Poland and at the Holycross Cancer Center in Kielce, Poland. Tumor stage was assigned according to the 2009 International Federation of Obstetrics and Gynecologists (FIGO) staging criteria for vulvar carcinoma. Patients with microscopically confirmed VSCC with no distant metastases at both early (54 FIGO stage I, 3 FIGO stage II) and advanced stages (32 FIGO stage III), operated between January 2002 and December 2017 were enrolled. Patients with non-squamous vulvar cancers were excluded. Median follow-up time of the enrolled patients, determined from the date of surgery to the date of death or the date of the last interview was 39 months (range 1–207 months). Adjuvant chemo- and/or radiotherapy as well as type and time of recurrences were registered. Additionally, ten patients with vulvar intraepithelial precancer lesions (eight with high-grade squamous intraepithelial lesions, HSIL and two with differentiated vulvar intraepithelial neoplasia, dVIN) and five with local recurrence were enrolled at the two participating centres, in order to examine whether the phenomena identified in primary VSCC are also of relevance at both early and late stages of vulvar carcinogenesis.

### DNA isolation and HPV genotyping

Tissue samples were frozen in liquid nitrogen immediately after collection and stored at − 70 °C until DNA isolation. The hematoxylin–eosin stained slides corresponding to the collected snap-frozen tissues were initially reviewed by the gynaecological pathologist (EB-Z), to whom the subsequent molecular and immunohistochemical results were blinded. DNA was isolated from approximately 50 mg of pulverised tumour samples (with Microdismembrator II, B Braun Biotech International, Melsungen, Germany) using the NucloSpin Tissue Kit (Macherey–Nagel, Düren, Germany), according to the manufacturer’s instructions. High-risk human HPV (hrHPV) status was determined using the AmpliSens HPV HCR-genotype-titre-FRT test (InterLabService Ltd., Moscow, Russian Federation), which detects 14 high risk HPV (hrHPV) genotypes, following the manufacturer’s instructions.

### 16S rRNA next generation sequencing

The 16S hypervariable regions were amplified using the 16S Ion Metagenomics Kit (Life Technologies, Grand Island, NY, USA). For each sample, two PCR reactions were prepared, one for each primer set (V2-4-8 and V3-6, V7-9) to amplify in total seven hypervariable regions (V2, V3, V4, V6, V7, V8 and V9) of bacterial gene coding 16S ribosomal RNA (16S rRNA) [14]. 16S rRNA is the component of the small subunit of the bacterial ribosome. The kit also includes additional primers allowing discrimination between species belonging to highly homologous genera such as *Lactobacillus* and *Bacteroides* that would be indistinguishable using just 16S rRNA gene primers.

After the PCR reaction, equal volumes of amplicons were pooled and then the amplification products were purified using CleanNGS (CleanNA, Waddinxveen, The Netherlands). The quantity of PCR product was measured using Qubit 2.0 (Invitrogen, USA) and dsDNA HS Assay Kit (Life Technologies, Grand Island, NY, USA) to calculate the input for library preparation. Subsequently, 60 ng of pooled amplicons were used to prepare the library with the Ion Plus Fragment Library Kit and Ion Xpress Barcode Adapters 1–96 Kit (Life Technologies, Grand Island, NY, USA) according to the manufacturer’s instructions. The products were purified again and then qPCR was performed with the Ion Universal Library Quantitation Kit (Life Technologies, Grand Island, NY, USA) using the QuantStudio 5 Real-Time PCR System (Applied Biosystems, USA). Real-time PCR results were used to determine the library dilution factor. Each sample was diluted to a concentration of 10 pM. Equal volumes of diluted samples were combined and added to the Ion Chef Instrument sample tube (Life Technologies, Grand Island, NY, USA) to prepare them for sequencing performed on the Ion GeneStudio™ S5 (Thermo Fisher Scientific, Waltham, MA, USA) using the Ion 520™ Chip Kit (Life Technologies, Grand Island, NY, USA). Base calling was performed using Torrent Suite version 5.12.1 (Life Technologies, Grand Island, NY, USA).

### Detection of microbes in VSCC samples by quantitative polymerase chain reaction (qPCR)

Custom Microbial DNA qPCR Array plates (Qiagen, Hilden, Germany) designed to target the 16 s rRNA gene were used to test for the following 12 bacterial species: *Lactobacillus crispatus* (cat. No. BPID00186A), *Lactobacillus gasseri* (BPID00189A), *Lactobacillus iners* (BPID00190A), *Prevotella bivia* (BPID00272A), *Prevotella disiens* (BPID00276A), *Fusobacterium nucleatum* (BPID00160A), *Staphylococcus aureus* (BPID00314A), *Gardnerella vaginalis* (BPID00163A), *Pseudomonas aeruginosa* (BPID00288A), *Atopobium vaginae* (BPID00041A), *Bacteroides fragilis* (BPID00051A) and *Mobiluncus curtisii* (BPID00219A) according to the manufacturer's instructions. Briefly, reactions were carried out in QuantStudio 5 (Applied Biosystems) in a final volume of 10 μl reaction mix that included 10 ng of template DNA. The baseline value for the cycle threshold (Ct) was set at 8 to 20 cycles and the threshold at 0.2. The ‘Custom Microbial DNA qPCR Array ID Data Analysis’ template (Qiagen) was used to determine whether the qPCR Ct values obtained for each target indicated a high positive (+), low positive (+/−) or negative (−) signal. Positive PCR control (PPC, cat. No. BPCL00365A), two positive bacterial control templates (Pan Bacteria 1, cat. No. BPCL00360A and Pan Bacteria 3, cat. No. BPCL00362A) and the negative control (nuclease-free water) were included in each run.

### Immunohistochemical staining

The selected proteins were immunohistochemically detected in formalin-fixed paraffin-embedded 4-μm sections of VSCC tumours. Specimens were deparaffinised with xylene and rehydrated with decreasing concentrations of ethanol (from 99 to 50%). Heat-induced epitope retrieval was carried out in Target Retrieval Solution (Dako, Glostrup, Denmark) for 30 min at 97 °C. After cooling, the slides were treated for 5 min with an endogenous peroxidase blocker (Dako).

The slides were then incubated with the following antibodies:

- polyclonal against Lipid A of lipopolysaccharide (LPS) (PA1-73,178, ThermoFisher Scientific), 1:50 dilution, 1 h at room temperature (RT),
- monoclonal against lipoteichoic acid (LTA) (MA1-40134, ThermoFisher Scientific), 1:50 dilution, 1 h at RT,
- polyclonal against CD66b (ab214175, Abcam), 1:100 dilution, 1 h at RT,
- polyclonal against CTSG (PA5-96373, ThermoFisher Scientific), 1:50 dilution, overnight at 4 °C
- polyclonal against ELANE (PA5-84738, ThermoFisher Scientific), 1:1000 dilution, 1 h at RT.
- polyclonal PRTN3 antibody (HPA005938, Sigma-Aldrich), 1: 200 dilution, 1 h at RT.

The sections stained with LPS were then incubated with horseradish peroxidase (HRP) labelled Donkey anti-Goat IgG secondary antibody (H + L) (PA1-28664, ThermoFisher Scientific) at 1:4000 dilution for 30 min in RT. The colour reaction products of LPS, LTA Cathepsin G, CD66b and neutrophil elastase stainings were developed with the Envision Detection System, EnVision FLEX+, Mouse, High pH Detection System (Dako). Nuclear contrast was achieved with 20 second counterstaining with haematoxylin (Dako). The analysis of the results was performed using optical microscopy (×400).

### Data processing and statistical analysis

Alpha diversity of microbial communities analysed by NGS was calculated using Shannon index with diversity function from vegan package (version 2.5-7) and displayed with R software (version 3.6.3). Hierarchical clustering was performed with hclust function from the stats package and visualized with heatmap.2 function from gplots package (version 3.1.1). Ward linkage was used for the clustering.

The presence/absence of bacterial species detected by qPCR in the examined groups of vulvar tissues was compared by Chi-square or the exact Fisher two-tailed tests. Chi-square testing power was examined with pwr library (version 1.3.0) in the R environment (version 4.1.1). Immunohistochemical results were compared in the sample groups using the Kruskal–Wallis test, followed by the Dunn multiple comparison post-test, and significant alterations were indicated by asterisks (*, p-value ≤ 0.05; **, p-value ≤ 0.01; ***, p-value ≤ 0.001; ****, p-value ≤ 0.0001). The progression was defined as local recurrence, regional lymph node recurrence, or cancer-related death. Progression-free survival (PFS) was determined from the date of surgery to the date of progression or the date of the last follow-up visit. Survival analysis was performed using a Kaplan‐Meier survival analysis and the statistical significance was determined by a log rank test (Mantel-Cox). These statistical analyses were carried out using GraphPadPrism 6 (San Diego, CA, USA). For all statistical analyses, the differences were considered statistically significant at p < 0.05.

## Results

DNA isolated from 56 VSCC primary tumours was subjected to next generation sequencing (NGS) of 18S rRNA. Two samples were excluded from further analysis because the patients were lost to follow-up. Additional five samples were eliminated due to the small number of sequence reads for the 18S rRNA (generating less than 500 reads per sample). The remaining 49 tumours were obtained from patients with median age of 71.2 years (rage 37.3–94.2) with VSCC at both early (29 FIGO stage I, 2 FIGO stage II) and advanced stages (18 FIGO stage III), and followed-up for a median of 36 months (range 1 to 133 months). The samples were obtained from patients who progressed (designated “progVSCC”, n = 24) and from those who were disease-free (“d-fVSCC”, n = 25) during follow-up. The samples were stratified as positive for hrHPV (hrHPV +, n = 27) and negative for hrHPV (hrHPV–, n = 20); in two tumours the status of hrHPV was not determined. In this sample set, NGS generated a median of 16,382 reads per sample (1Q–3Q: 9491–25014). A median of 41 operational taxonomic units (OTUs) per sample was observed. The median number of OTUs was lower in progVSCC tumours than in d-fVSCC tumours (40.50 vs 44, respectively) and equal (40) in tumours positive and negative for hrHPV.

The hierarchical clustering based on the abundance of family (Additional file 1: Fig. S1) and genera or species (data not shown) in VSCC did not indicate specific clusters for either clinical tumour types (d-fVSCC and progVSCC) or the status of tumour hrHPV. Our results also showed no significant differences between the two clinical tumour types or hrHPV statuses in overall alpha diversity, as measured by the Shannon index, which reflects species numbers and evenness of species abundance (Additional file 2: Fig. S2).

**Fig. 1:**
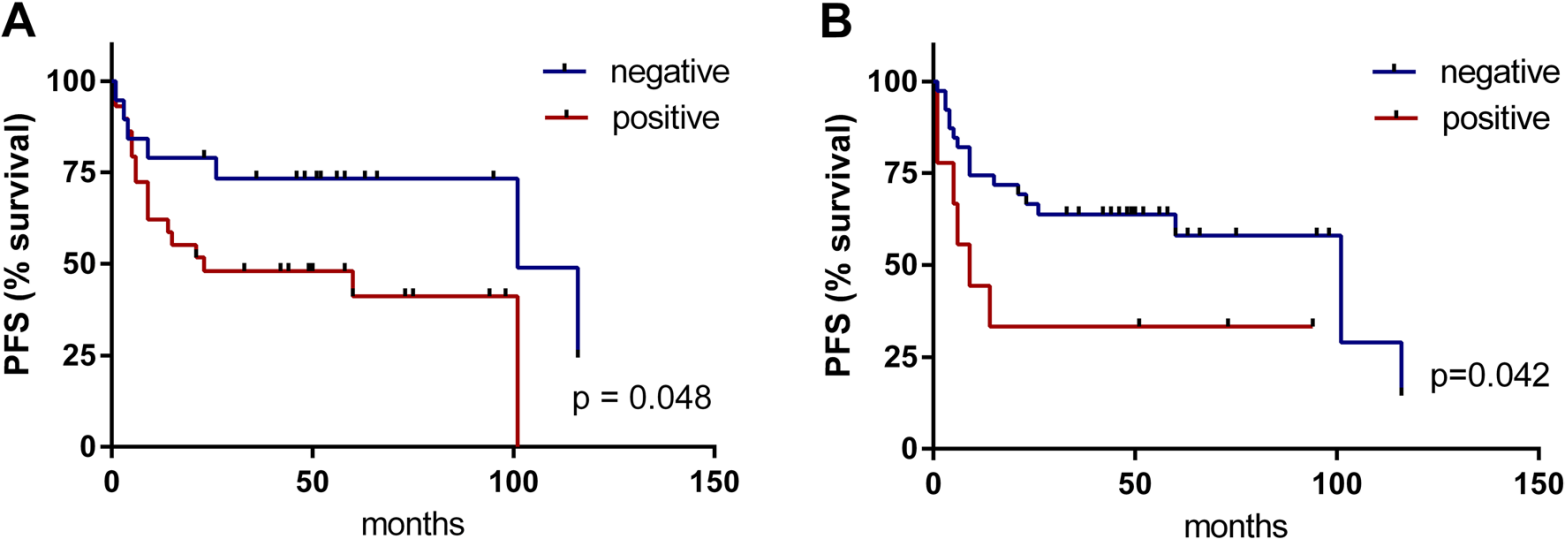
Survival curves (time to progression) of patients with primary VSCC (n = 48) according to the presence of *Fusobacterium nucleatum* (**A**) and *Pseudomonas aeruginosa* (**B**) in tumours. *PFS* progression-free survival

Based on a combination of next-generation sequencing data on the abundance of bacterial species in VSCC obtained in this study with the results of our previous work [13] and a review of literature data on bacteria in the female reproductive tract linked to gynaecological cancers [15], we selected 12 bacterial targets to be analysed in a qPCR array. The presence of these species in vulvar tissue samples was examined in patients with premalignant vulvar lesions (HSIL, n = 7 and dVIN, n = 2), primary VSCC (n = 48, followed-up for a median of 39 months), and healthy women (n = 12) who underwent routine plastic surgery of the reproductive organ, as described previously [13]. The Chi-square test revealed a statistically significant difference between the results obtained (high positive and low positive *versus* negative qPCR signal) in the three sample groups for two bacterial species, namely *Fusobacterium nucleatum* and *Mobiluncus curtisii* (p = 0.005 and 0.031 respectively). The relatively low sample number, especially of control samples, was found sufficient to detect large effects (w = 0.5) with high power (pwr = 0.85) and medium effects (w = 0.3) with medium power (pwr = 0.44). Next, we looked at the survival curves of patients with primary VSCC tumours classified by the high-positive *versus* low-positive and negative qPCR signal for the bacterial species analysed. The main clinical data of these patients are provided in Additional file 6**:** Table S1. The abundance of *Fusobacterium nucleatum* and *Pseudomonas aeruginosa* was found to be related to a shorter time to progression (23 vs 101 and 9 vs 101 moths, respectively) (Fig. 1). Fisher’s exact two-tailed test revealed no statistical differences between the groups positive for hrHPV (n = 27) and negative for hrHPV (n = 20) of VSSC tumours in terms of the presence of 12 bacterial species examined.

In order to visualize intratumoral bacteria populations, we performed IHC staining using antibodies against bacterial lipopolysaccharide (LPS) and lipoteichoic acid (LTA) to detect Gram-negative and Gram-positive bacteria, respectively. Due to the particle sizes stained with LPS and LTA (see Additional file 3: Fig. S3), we assumed that the staining images were of phagocytised bacterial components rather than live bacteria. To verify neutrophil activation, the most abundant phagocytes in humans known for their ability to engulf and eliminate bacterial pathogens, we performed neutrophil IHC staining with anti-CD66b antibody, as well as main neutrophil serine proteases’ (NSP) staining with anti -CTSG (cathepsin G), -ELANE (neutrophil elastase) and -PRTN3 (proteinase 3) antibodies in VSCC samples. These NSPs produced by activated neutrophils at inflammation sites play an important role in immune protection against bacterial threats [16].

**Fig. 2:**
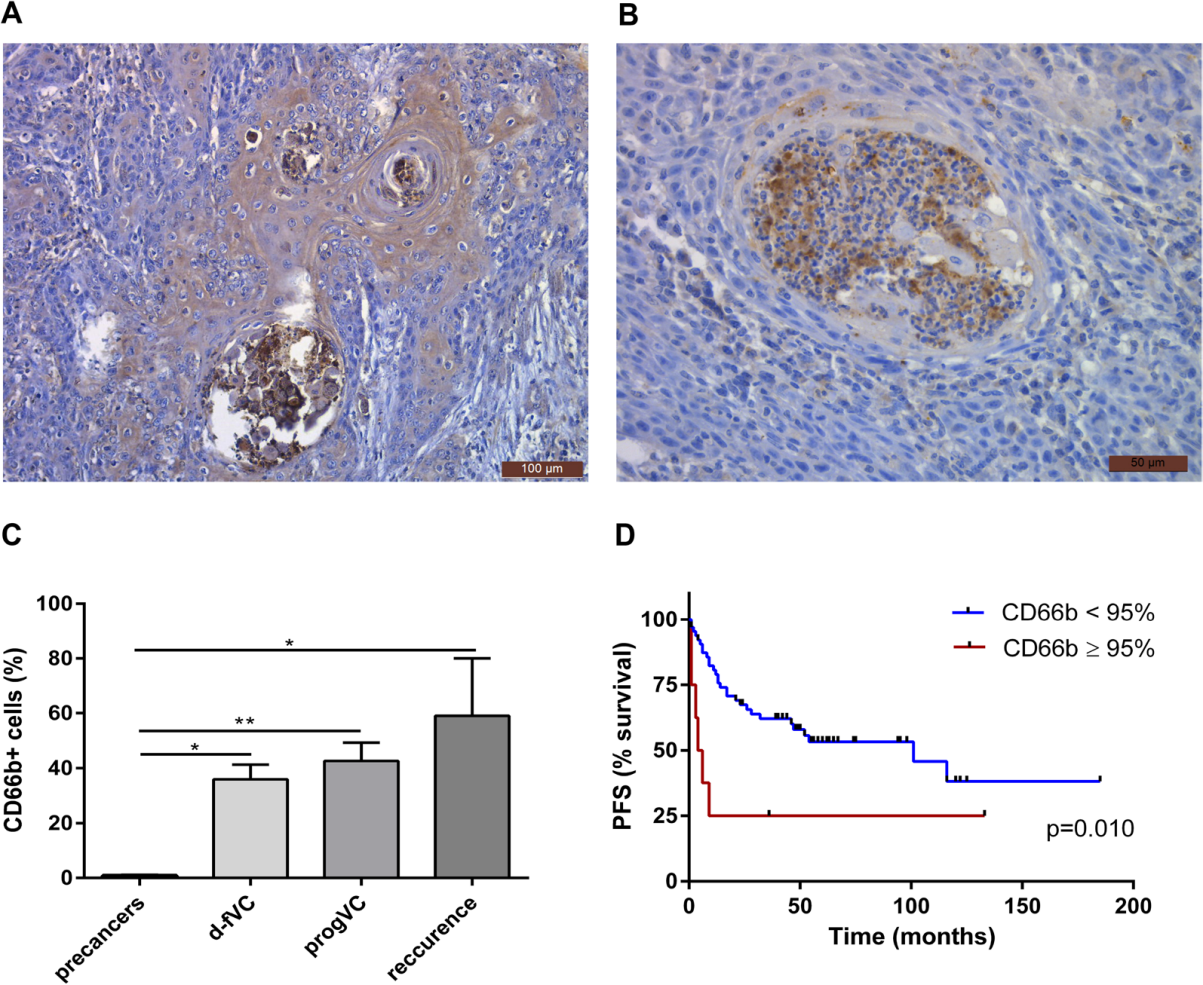
Neutrophils in precancer lesions and VSCC tumours. Examples of immunohistochemical CD66b staining performed on tissue sections of VSCC tumours; images taken at × 20 **A** and × 40 **B** magnifications. The percentage of CD66b + neutrophils (**C**), in samples of vulvar precancer (HSIL, n = 7; dVIN, n = 1), d-fVSCC (n = 39), progVSCC (n = 29) and vulvar recurrence (n = 4). Survival curve (time to progression) according to the proportion of CD66b + neutrophils (**D**) in primary VSCC tumours (n = 67). *PFS* progression-free survival; < 95% and ≥ 95%, proportions of CD66b + neutrophils

Cytoplasmic CD66b staining was found in both scattered tumour-associated neutrophils (TANs) and TANs in circumscribed aggregates of white blood cells, termed microabscesses (visible in Fig. 2B). The observed numbers of CD66b-positive TAN suggest their increased count during VSCC progression (Fig. 2C). The survival curves of the two groups of patients with primary VSCC tumours classified by the percentage of CD66b + neutrophils are shown in Fig. 2D. Median disease-free survival rates were 101 and 5 months in patients with low and higher percentages of CD66b + neutrophils, respectively.

**Fig. 3:**
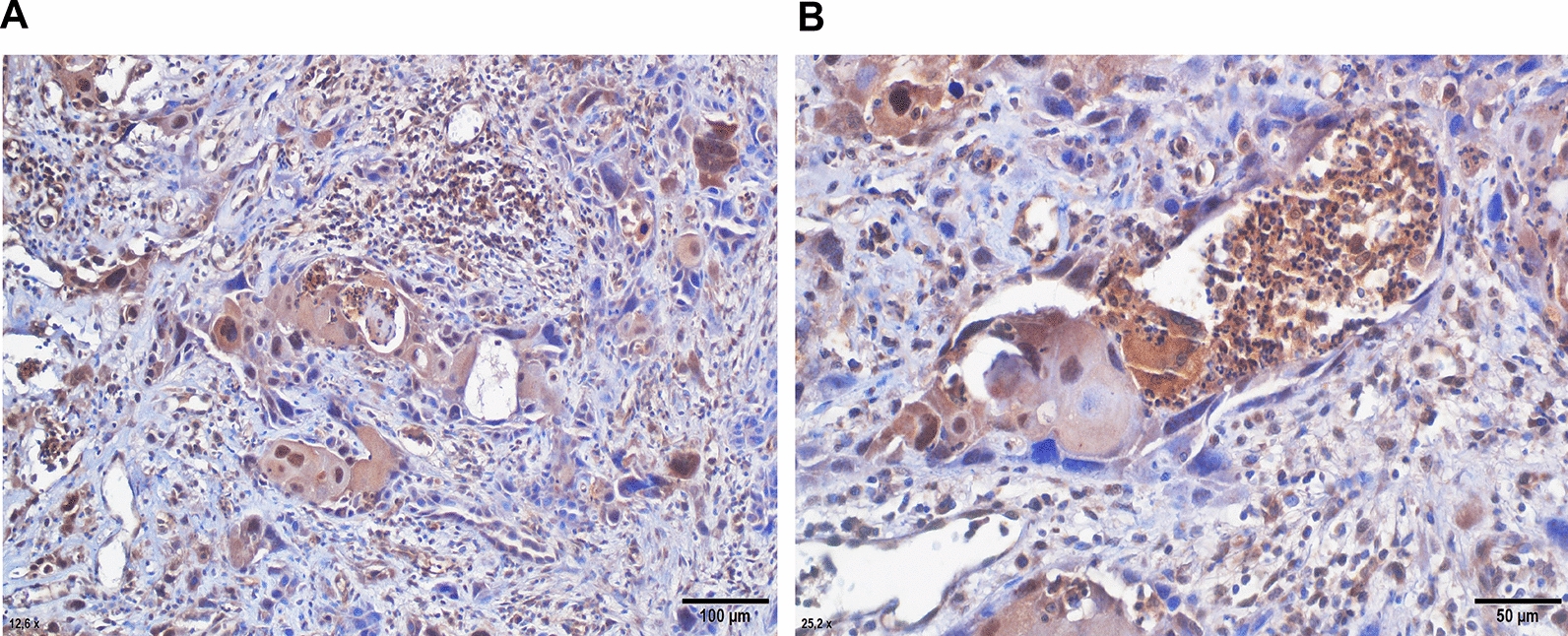
Examples of immunohistochemical CTSG staining performed on tissue sections of VSCC tumour. CTSG positive inflammatory cells in the stroma (**A**) and within the microabscess (**B**). Images taken at × 20 (**A**) and × 40 (**B**) magnifications

Immunohistochemical staining demonstrated the presence of CTSG in virtually all examined VSCC samples (Fig. 3). This neutrophil serine protease was observed in all neutrophils and, to a lesser extent, in plasmocytes and macrophages. Granular cytoplasmic positivity for cathepsin G in neutrophils was localised to the tumour surface, microabscesses, or histological ulceration. Semiquantitative CTSG scoring (0–3) suggested an increase in neutrophil counts and a decrease in plasmocyte counts during the course of VSCC, however, this observation did not reach statistical significance (Additional file 4: Fig. S4).

**Fig. 4:**
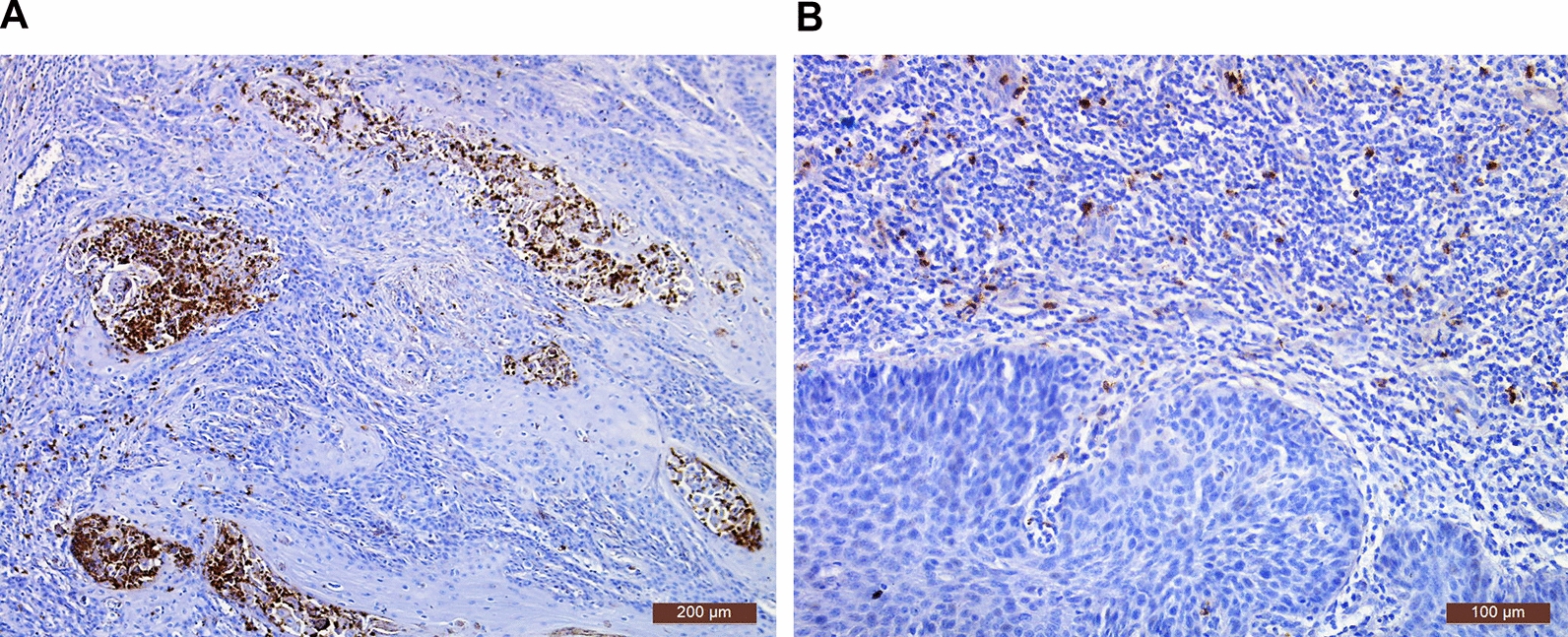
ELANE-positive neutrophil images within exemplary VSCC tumour specimens. ELANE staining in cells in the stroma (**A**) and within microabscess (**B**). Clusters of neutrophils within cancer foci (**A**) and inflammatory infiltrates (**B**). Images taken at × 10 (**A**) and × 20 (**B**) magnifications

Granular cytoplasmic staining of ELANE (Fig. 4) was found in neutrophils within cancer crypts, ulcers, inflammatory infiltrates and microabscesses in most of the tumours examined (89% of primary VSCC, 48/54). Although not statistically significant, there was a notion, based on the semiquantitative ELANE score (0–3) in the examined sample set, that the number of ELANE + neutrophils may increase during vulvar carcinogenesis (Additional file 1: Fig. S5).

**Fig. 5:**
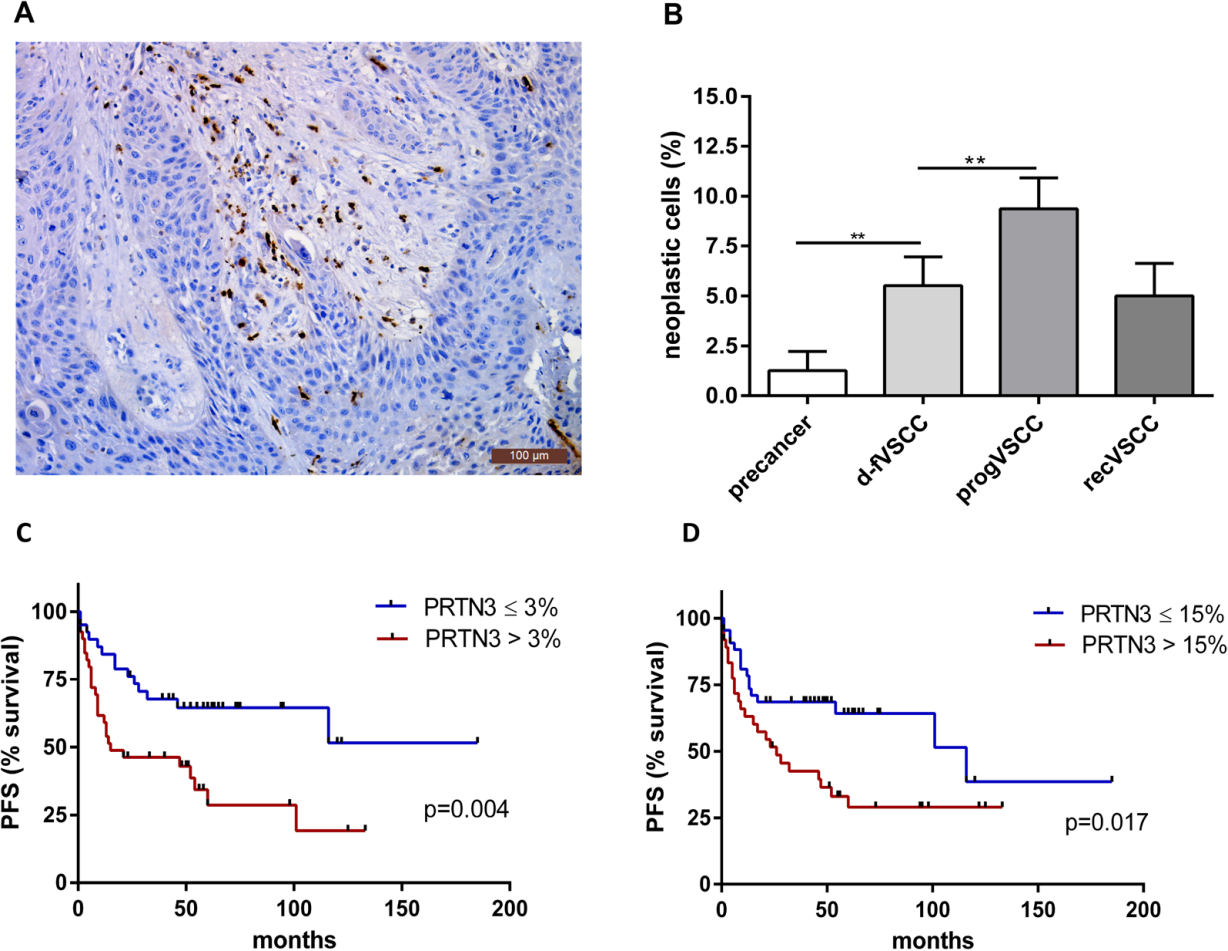
Immunohistochemical analysis of PRTN3 in VSCC. An example of PRTN3 staining performed on the tumour tissue section, image taken at × 20 magnification (**A**). The percentage of PRTN3 positive neoplastic cells in vulvar precancers (HSIL; n = 6 and dVIN; n = 2), d-fVSCC (n = 42), progVSCC (n = 40) and recurrent VSCC (n = 4) (**B**). Survival curves (time to progression) according to the proportion of PRTN3 positive cancer cells (**C**) and macrophages (**D**) in primary VSCC tumours (n = 64). *PFS* progression-free survival, ≤ 3% and > 3%, proportions of PRTN3 + cancer cells, ≤ 15% and > 15%, proportions of PRTN3 + macrophages

**Fig. 6:**
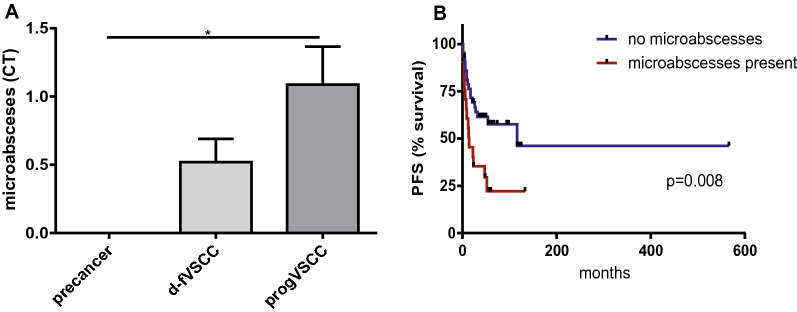
Tissue microabscesses in vulvar precancers and VSCC. Numbers in precancer tissues (HSIL, n = 5; dVIN, n = 2) and tumour tissues (d-fVSCC, n = 32; progVSCC, n = 30) (**A**). Survival curves (time to progression) according to the number of microabscesses (**B**) in primary VSCC tumours (n = 64). *CT* microabscesses number per low power field (mean of three counts), *PFS* progression-free survival

Cytoplasmic staining of PRTN3, the last of the neutrophil serine proteases examined, was observed in neoplastic cells, macrophages, and lymphocytes as previously described [13] (Fig. 5). A major subset of PRTN3 stained VSCC samples (n = 64) was taken from that study. The survival curves of patients with primary VSCC tumours categorised by the number of PRTN3 positive cancer cells (undefined PFS vs median disease-free survival of 15 months in patients with low and higher percentage of PRTN3-positive cells, respectively) and macrophages (median disease-free survival of 116 vs 26 months in patients with low and higher percentage of PRTN3-positive cells, respectively) are shown in Fig. 6C and D.

Finally, our attention was drawn to the histological microabscesses in VSCC. We observed well-developed microabscesses separated from the surrounding tissue by a membrane. The stainings of CD66b and NSP within the microabscess confirmed the accumulation of neutrophils, a histological hallmarks of neutrophilic microabscesses. The number of microabscesses within tumours appeared to increase during disease progression and, importantly, was correlated with the time to progression (Fig. 6). Patients with primary VSCC with no microabscesses had a median disease-free survival of 116 months as compared to 14 months in a group with higher count of microabscesses in the tumours.

## Discussion

Current knowledge on the molecular biology of VSCC is scarce. In recent years, new treatments that could exploit the virology of HPV and/or enhance the host immune response have been proposed, but there is still a paucity of clinical trials in VSCC [17]. Additionally, the individualisation of currently available treatment modalities is hampered by the lack of reliable prognostic factors approved for clinical practice. The FIGO staging was shown not to be fully correlated with the prognosis of patients with VSCC [18]. Even the tumour-free pathological resection margin at a distance of < 8 mm, long considered a significant risk factor for vulvar recurrence, has been challenged as associated with local recurrence [19]. Patients with multiple positive regional lymph nodes benefit from adjuvant radiation therapy after surgery, but in patients with a single positive lymph node, the survival benefit still remains debatable [20–22]. Likewise, the role of adjuvant chemoradiotherapy in patients with lymph node involvement, as well as of neoadjuvant treatment modalities in advanced cases of VSCC, is not well defined.

Previously, we have reported that microflora alterations can contribute to vulvar cancer progression [13], suggesting new approaches to be considered in the development of therapies for patients with VSCC. Previous studies on cervical cancer revealed a causal link between vaginal dysbiosis and cervical oncogenesis, as well as the notion that dysbiosis constitutes a risk factor for both HPV acquisition and persistence [15, 23, 24]. In this study, our objective was to identify vulvar intratumour bacteria that could contribute to cancer progression. However, using 16S rRNA next generation sequencing, we did not determine significant changes in the composition of bacterial families, genera or species that would certainly be associated with the progression of VSCC. Neither the bacterial composition was clearly distributed by the status of the hrHPV infection. However, in a targeted analysis of the 12 bacterial species by qPCR, the presence of *Fusobacterium nucleatum* and *Pseudomonas aeruginosa* was revealed to be related to a shorter time to disease progression in patients with VSCC. *F. nucleatum*, an anaerobic Gram-negative bacterium, inhabits the oral cavity and the gastrointestinal and genital tracts. *F. nucleatum* was reported to be overabundant in several types of cancers and to promote metastasis [25]. The high intratumoral burden of *F. nucleatum* corresponds to poor survival rates in patients with cervical carcinoma [26]. Intriguingly, this high burden has been reported to occur mutually exclusive to HPV infection in head and neck tumours [27]. In the mouse model of colorectal neoplasia, *Fusobacteria* were shown to recruit tumour-infiltrating myeloid cells and generate a pro-inflammatory microenvironment conducive to progression [28]. *P. aeruginosa*, another bacterium that, as our study suggests, contributes to the progression of VSCC, is a ubiquitous aerobic Gram-negative bacterium and an opportunistic pathogen [29]. Many anaerobic bacterial species, including facultatively anaerobic *P. aeruginosa*, infiltrate the hypoxic regions of tumours, hence the notion of bacteria-mediated cancer treatment [30]. In a study of the cervical microbiota in different HPV infection statuses in cytologically normal women, *F. nucleatum* was found to be associated with baseline HPV positivity, while *P. aeruginosa* had a relatively higher abundance in HPV-negative women [31]. Our data (not shown) did not reveal associations between hrHPV status and the presence of these two bacterial species in vulvar tumours.

Neutrophils are the first cells to respond to inflammatory or infectious conditions. After trafficking into tissues, neutrophils promote the clearance of extracellular bacterial pathogens. During inflammation, activated neutrophils release neutrophil serine proteases (NSPs) that contribute to antibacterial responses. Neutrophils affect carcinogenesis, however, because of their plasticity and heterogeneity they may play both protective and damaging roles, including the acceleration of metastatic spread [32–34]. The accumulation of TAN has been associated with an unfavourable prognosis in many cancers [35]. Our study adds to the evidence that TANs, known to promote cancer progression, are also associated with VSCC carcinogenesis. A high abundance of CD66b, the neutrophil activation marker, was found to be associated with poor survival in our cohort of patients with VSCC, suggesting that neutrophils polarise towards a pro-tumorogenic N2-like phenotype in VSCC.

Neutrophils are the main type of immune cells that, following infection, participate in the formation of microabscesses. In a recent analysis of the proteomic components of different abscess regions [36], neutrophil surface markers and multiple neutrophil-specific antimicrobial factors—including CTSG and ELANE—were detected in the abscess at the host–pathogen interface. We found that microabscesses can occur in VSCC and that their increased counts correlate with disease progression. It can be hypothesised that acquired N2-like phenotype TANs exhibit impaired bactericidal activity and are permissive for the survival of vulvar cancer cells. In addition, these mechanisms may be fuelled by multiple bacterial strategies to counteract antimicrobial immune functions. These strategies include manipulation of neutrophil degranulation [37]. Indeed, both *F. nucleatum* and *P. aeruginosa* were confirmed to mediate the degranulation processes and release of ELANE [38, 39].

As granular neutrophil components, NSPs are known to play key antibacterial roles in phagocytosis, exocytosis (degranulation), and NETosis (release of extracellular traps by neutrophils) [16]. However, accumulating evidence shows that a variety of neutrophil effector mechanisms, on top of those of NSPs, may be exploited for tumour promotion [34]. We have shown the main neutrophil serine proteases: CTSG, ELANE, and PRTN3 in VSCC specimens. In this study, the proportion of PRTN3-positive cancer cells and macrophages was associated with a shorter time to progression in patients with VSCC). In summary, our data underline an important role for bacterial colonisation in the promotion of VSCC by disturbing the interactions between infiltrating immune cells, especially neutrophils, and tumour cells.

## Conclusions

In conclusion, although the NGS results did not support the notion of global changes in the VSCC microbiota to participate in cancer promotion, based on our targeted bacterial detection, we postulate that *Fusobacterium nucleatum* and *Pseudomonas aeruginosa* species might enhance vulvar carcinoma progression. Yet, further research, with larger independent sample sets, is warranted to verify this assumption. We demonstrated that neutrophilic inflammation in the VSCC environment is permissive of tumour colonising bacteria and supports cancer progression. This study reveals targeting neutrophils as a new possible treatment option to be developed for VSCC patients. Potential approaches include blocking neutrophil recruitment to tumours, activating antitumor or silencing protumour neutrophil functions through multiple means, such as targeting neutrophil extracellular traps’ (NET) formation mechanisms or harnessing granule components, such as NSPs [35, 40].

## Supplementary Information

**Additional file 1: Fig. S1**. Comparison of the VSCC microbiota at the family level using heat-map analysis. The progVSCC and d-fVSCC samples are depicted in the top line in brown and turquoise colours, respectively. The hrHPV + and hrHPV− sample statuses are marked in red and purple, respectively. The relative abundance of each bacterial family was also represented by a colour; red indicates a high proportion and blue indicates a low abundance. Hierarchical clustering of the VSCC microbiota was performed using the Ward linkage of the upper quartile of the most represented OTUs.

**Additional file 2: Fig. S2**. Analysis of the alpha diversity in d-fVSCC compared to progVSCCand hrHPV- compared to hrHPV + VSCC tumours.

**Additional file 3: Fig. S3**. LPSand LTAstaining of VSCC tumours. Images taken at × 40 magnification.

**Additional file 4: Fig. S4**. Semiquantitative CTSG scoring results in neutrophilsand plasmocytesin vulvar precancers, d-fVSCC, progVSCCand recurrent VSCC.

**Additional file 5: Fig. S5**. Semiquantitative ELANE scores in neutrophils in vulvar precancers (HSIL; n = 5 and dVIN; n = 2), d-fVSCC (n = 31), progVSCC (n = 31) and recurrent VSCC (n = 5).

**Additional file 6: Table S1**.

## Data Availability

Not applicable.

## Abbreviations

Ct: Cycle threshold
CTSG: Cathepsin G
dVIN: Differentiated vulvar intraepithelial neoplasia
ELANE: Neutrophil elastase
FIGO: The International Federation of Obstetrics and Gynecologists
hrHPV: High risk HPV
HPV: Human papillomavirus
HSIL: High-grade squamous intraepithelial lesions
LPS: Lipopolysaccharides
LTA: Lipoteichoic acid
NET: Neutrophil extracellular trap
NETosis: Release of extracellular traps by neutrophils
NGS: Next generation sequencing
NSP: Neutrophil serine protease
OTU: Operational taxonomic units
PFS: Progression-free survival
pwr: Power
PRTN3: Proteinase 3
qPCR: Quantitative polymerase chain reaction
TAN: Tumour-associated neutrophil
w: Effect size
VSCC: Vulvar squamous cell carcinoma
progVSCC: Patients who progressed
d-fVSCC: Disease-free patients during follow-up
