# Supplementary figures and images for: The intratumour microbiota and neutrophilic inflammation in squamous cell vulvar carcinoma microenvironment
Source: J Transl Med. 2023 Apr 28;21:285. doi: 10.1186/s12967-023-04113-7 (PMC10141905; doi:10.1186/s12967-023-04113-7)

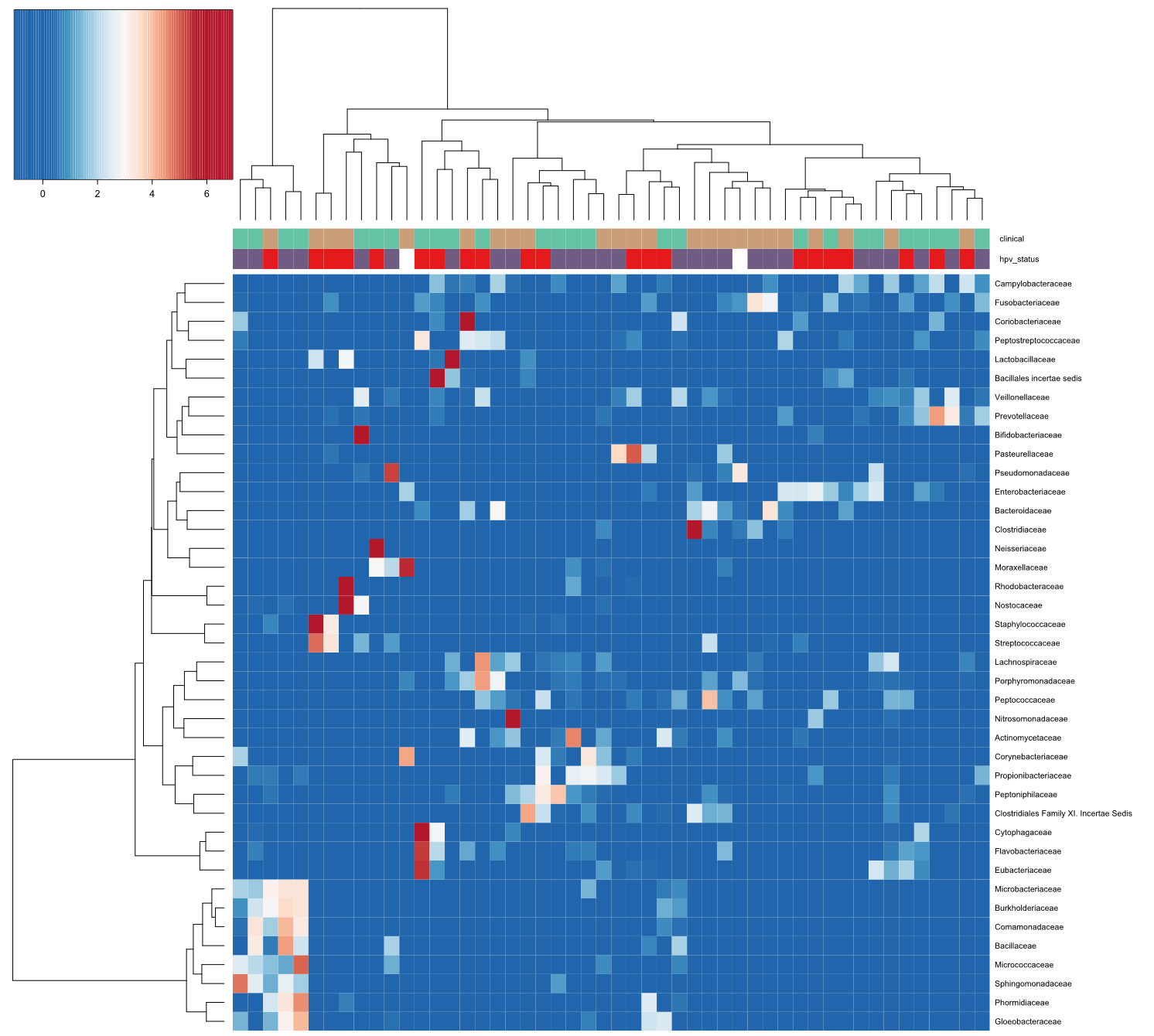

Supplement: Supplementary file 1 — Additional file 1: Fig. S1. Comparison of the VSCC microbiota at the family level using heat-map analysis. The progVSCC and d-fVSCC samples are depicted in the top line in brown and turquoise colours, respectively. The hrHPV + and hrHPV− sample statuses are marked in red and purple, respectively. The relative abundance of each bacterial family was also represented by a colour; red indicates a high proportion and blue indicates a low abundance. Hierarchical clustering of the VSCC microbiota was performed using the Ward linkage of the upper quartile of the most represented OTUs. [file 12967_2023_4113_MOESM1_ESM.jpg]

**A**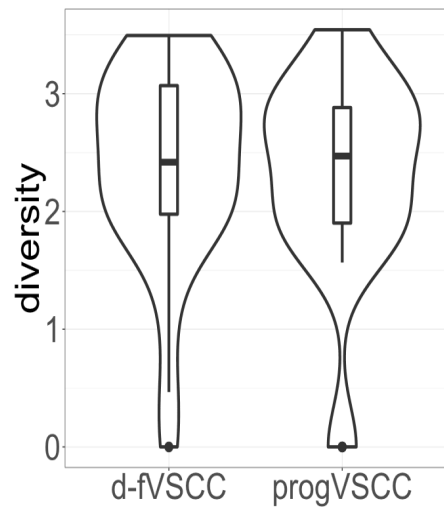**B**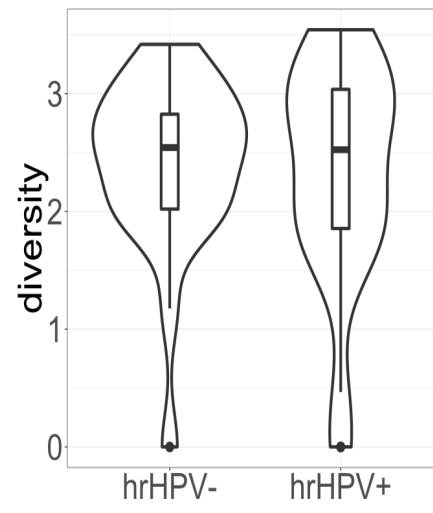

Supplement: Supplementary file 2 — Additional file 2: Fig. S2. Analysis of the alpha diversity in d-fVSCC compared to progVSCCand hrHPV- compared to hrHPV + VSCC tumours. [file 12967_2023_4113_MOESM2_ESM.pdf]

**A**

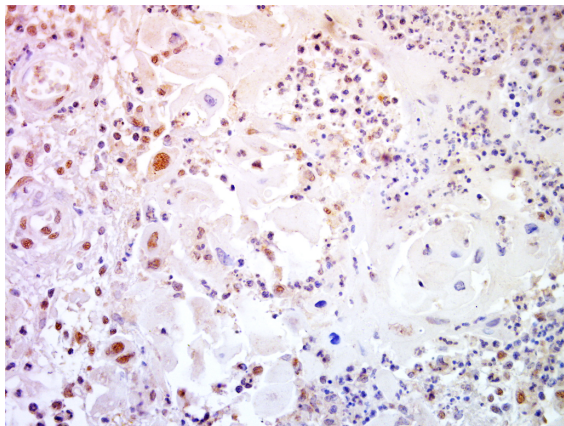

**B**

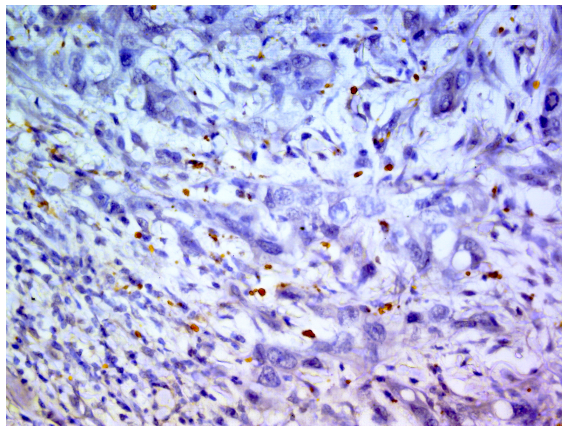

Supplement: Supplementary file 3 — Additional file 3: Fig. S3. LPSand LTAstaining of VSCC tumours. Images taken at × 40 magnification. [file 12967_2023_4113_MOESM3_ESM.pdf]

**A**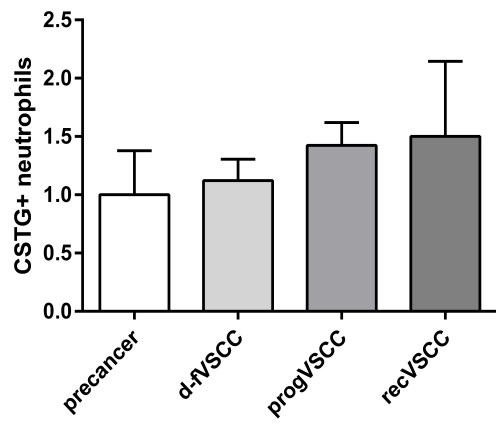**B**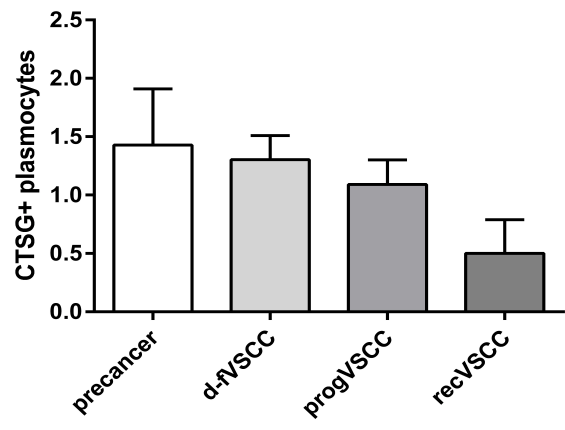

Supplement: Supplementary file 4 — Additional file 4: Fig. S4. Semiquantitative CTSG scoring results in neutrophilsand plasmocytesin vulvar precancers, d-fVSCC, progVSCCand recurrent VSCC. [file 12967_2023_4113_MOESM4_ESM.pdf]

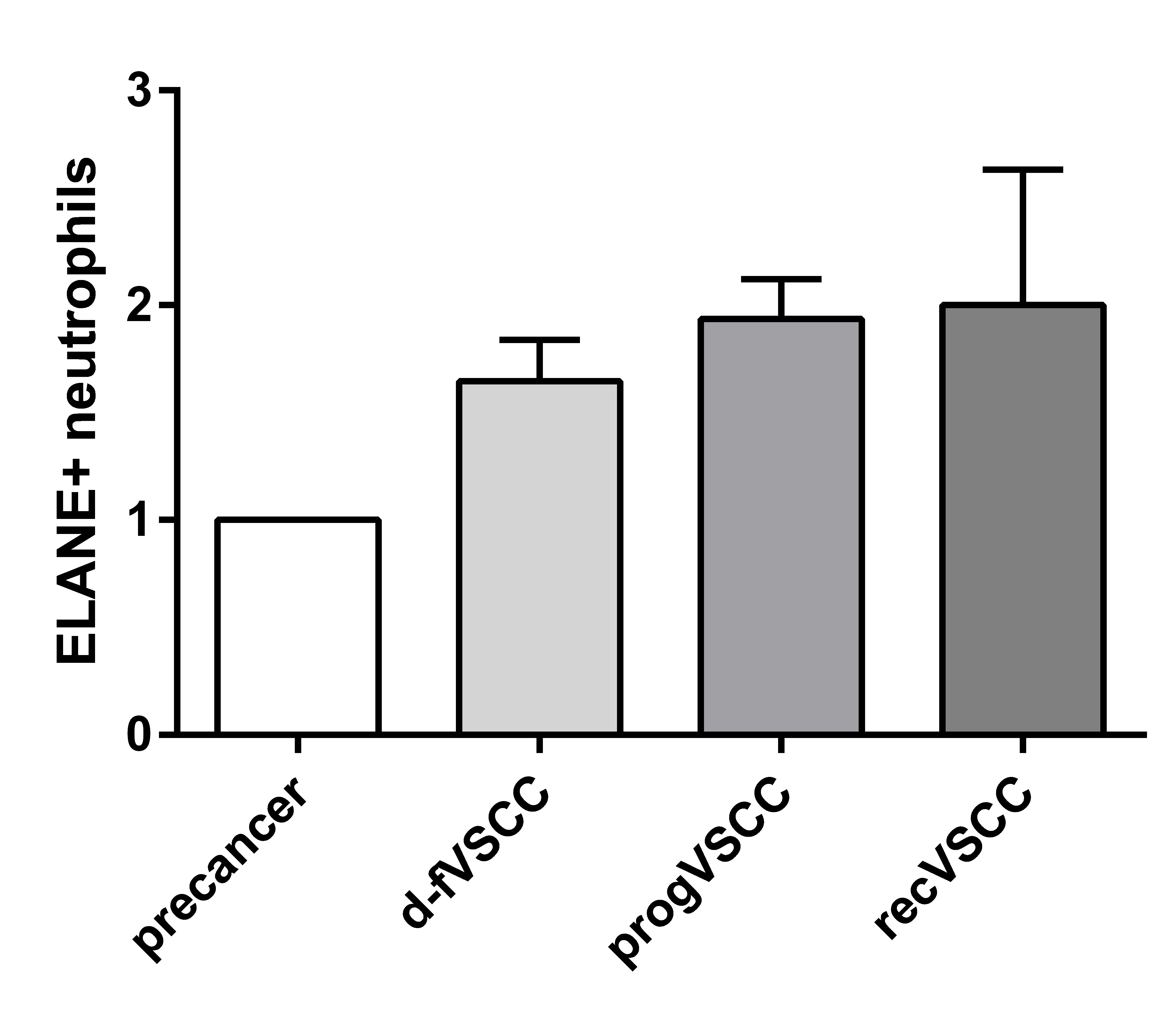

Supplement: Supplementary file 5 — Additional file 5: Fig. S5. Semiquantitative ELANE scores in neutrophils in vulvar precancers (HSIL; n = 5 and dVIN; n = 2), d-fVSCC (n = 31), progVSCC (n = 31) and recurrent VSCC (n = 5). [file 12967_2023_4113_MOESM5_ESM.jpg]
